# A Two-Year Retrospective Study of Blood Cultures in a Secondary Western Greece Healthcare Setting

**DOI:** 10.3390/microorganisms14010107

**Published:** 2026-01-04

**Authors:** Eirini Tsolakidou, Ioannis Angelidis, Apostolos Asproukos, Aikaterini Chalmouki, Nikolaos Zalavras, Kyriakos Louca, Panagiota Spyropoulou, Aliki Markopoulou, Eleni Katsorida, Paraskevi Stathakopoulou, Konstantina Filioti, Dimitrios Markopoulos, Konstantina Tsitsa, Charalampos Potsios, Konstantinos Letsas, Panagiota Xaplanteri

**Affiliations:** 1 Department of Microbiology, General Hospital of Eastern Achaia, 25100 Aigio, Greece; 2 Department of Radiology, General Hospital of Eastern Achaia, 25001 Kalavrita, Greece; angelidisj.dynamics@gmail.com; 3 Infection Preventionist Nurse, General Hospital of Eastern Achaia, 25100 Aigio, Greece; 4 Department of Internal Medicine, General Hospital of Eastern Achaia, 25100 Aigio, Greece; 5 Department of Internal Medicine, University General Hospital of Patras, 26504 Rio, Greece

**Keywords:** blood culture, contaminants, retrospective study, ciprofloxacin resistance

## Abstract

Blood culture remains the gold standard for identifying bloodstream infections caused by bacteria and fungi. Isolation of the culprit microorganism onto agar plates also facilitates antimicrobial susceptibility testing. The purpose of this study was to determine the contamination rates, pathogen profile, and antimicrobial resistance in a secondary healthcare setting in a two-year timeframe. In this study, data regarding blood cultures of the years 2023 and 2024 were retrospectively analyzed to address the above questions. Blood cultures were incubated for seven days before being discarded as negative. The percentage of positive blood cultures for both years was 14.3%. Most positive cultures contained Gram-positive cocci, with a prevalence of coagulase-negative *Staphylococci*. In descending order, 72.72% were coagulase-negative *Staphylococci*, 15.15% were *Staphylococcus aureus*, and 12.12% were *Streptococci*. One strain of *S. aureus* was methicillin-resistant (MRSA), and one strain of *Enterococcus faecium* was vancomycin-resistant (VRE). Of the Gram-negative rods, 78.3% were Enterobacterales. Of these, *Escherichia coli*, *Klebsiella pneumoniae,* and *Proteus mirabilis* were the top pathogens. The remainder comprised eight strains of *Pseudomonas aeruginosa*, four strains of *Acinetobacter baumannii* (one pandrug-resistant), three strains of *Stenotrophomonas maltophilia*, one strain of *Sphingomonas paucimobilis*, and one strain of *Campylobacter jejuni*. The isolated fungi comprised *Candida parapsilosis*, *Candida glabrata*, and *Candida tropicalis*. Of the isolated *Escherichia coli* strains, 39.5% were resistant to ciprofloxacin regardless of origin (outpatient or hospitalized patients). Outpatient samples were taken in a Hemodialysis Unit that collaborates with our laboratory, obtained from patients with fever or other signs of infection. Distinguishing true bacteremia from contamination remains challenging. The contamination rate in our study was quite high at 5.3%. Since there is no dedicated phlebotomy team in our healthcare setting, in light of our results, educational courses have been conducted to demonstrate the best practices for sample collection.

## 1. Introduction

Blood culture remains the gold standard for identifying bacteria and fungi involved in bloodstream infections, a major cause of death both in the community and in hospitalized patients worldwide [1]. True bacteremia from contamination needs elucidation in many cases. Contamination leads to positive blood cultures that generate problems for both clinicians and laboratory staff [2]. Resistance to antibiotics of the isolated strains is also of importance and should be stated. Studies to further analyze and collect data regarding blood culture isolates in a hospital without a dedicated phlebotomy team are important to monitor and elucidate methodology procedures. The aim of this study was to determine the contamination rate, pathogen profile, and antimicrobial resistance in a secondary healthcare setting. To address the above questions, blood cultures from both hospitalized patients and outpatients taken in the years 2023 and 2024 were retrospectively analyzed.

## 2. Materials and Methods

Blood cultures taken in the years 2023 and 2024 at the Biopathology/Biochemistry (Microbiology) department of our hospital were retrospectively analyzed. The demographic data of the patients, including gender and department of origin, were also included. The departments of origin were the Emergency Department, Internal Medicine Ward, Orthopedics, Surgery, Cardiology, and a Hemodialysis Unit in collaboration with our laboratory. Blood culture bottles were inoculated into Aerobic Culture Bottle FA (Resin) and Anaerobic Culture Bottle FN (Resin), Autobio Diagnostics Co., Ltd. (Groenenborgerlaan 16, 2610, Antwerpen, Belgium). The identification of the isolated microorganism and antibiotic susceptibility testing was performed by the Vitek^®^ 2 Advanced Expert System (bioMerieux SA., 376, Chemin de l’ Orme 69280, Marcy-l’ Etoile, France). Further susceptibility testing, when needed, was performed by the disk diffusion method and/or a gradient method (E-test) according to EUCAST guidelines [3].

Statistical analyses were conducted using Microsoft Excel (Microsoft, Redmond, WA, USA) and PSPP (GNU PSPP, version v2.0.0, Free Software Foundation, Boston, MA, USA). Chi-square tests were performed using the SciPy package in Python (version 3.11; Python Software Foundation, Wilmington, DE, USA). Comparisons of categorical variables between groups (sex distribution across years, origin of patients, and antimicrobial resistance rates of *E. coli* and coagulase-negative *Staphylococci*) were performed using Chi-square (χ^2^) tests or Fisher’s exact tests where appropriate. Two-sided comparisons with a *p*-value < 0.05 were considered statistically significant.

The criteria for ordering a blood culture were not always defined. Systemic inflammatory response syndrome (SIRS) criteria and qSOFA (quick Sequential Organ Failure Assessment) were used for the samples taken in the Emergency Department and from hospitalized patients. No such data was available for the samples taken in the Hemodialysis Unit.

Contaminants were considered: coagulase-negative *Staphylococci* (CoNS) in a single bottle, skin bacteria without clinical criteria, and CoNS isolated from a single set of aerobic and anaerobic blood culture but not isolated from other possible sites of infection, e.g., urine, wounds, or central vein catheters. The contamination rate was calculated as the percentage of total blood cultures. True bacteremia due to CoNS was considered when the same phenotypic strain—that is, a strain with the same biochemical reactions and antibiotic susceptibility patterns—was isolated from more than one sample or was also isolated from different sites of infection. In our hospital, there is no dedicated phlebotomy team; therefore, it was a challenge to elucidate the procedures followed for blood collection and blood inoculation in the bottles. Although there are protocols for standard operating procedures in our hospital regarding the type of skin antiseptic, the volume drawn, and the time from obtaining the sample to its transport to the laboratory, these protocols were not consistent. For skin preparation, chlorhexidine was recommended in our hospital’s procedure for collecting blood cultures.

## 3. Results

In 2023, a total of 544 sets of blood cultures were cultivated, of which 386 corresponded to male patients and 158 to female patients; 163 samples were taken in the clinical wards and 381 in the Emergency Department and Outpatient Department. In 2024, a total of 746 sets of blood cultures were cultivated: 441 from male patients and 305 from female patients. The origin of the samples was 318 from the clinic wards and 428 from the Emergency Department and Outpatient Department. A total of 1290 blood culture sets were analyzed over the two-year period: 698 were taken in the Emergency and Outpatients departments, 401 were taken in the Internal Medicine ward, 111 were taken in the Hemodialysis Unit, 52 were taken in the Surgery Department, 18 were taken in the Orthopedics Department, and 10 were taken in the Cardiology Department.

In 2023, 61 (11.2%) of these blood cultures were positive: 36 from male patients and 25 from female patients. In 2024, 123 sets of blood cultures were positive (16.5%): 71 derived from male patients and 52 from female patients. The percentage of positive blood cultures for both years was 14.3%. In 2023, 43 positive cultures were taken in the Outpatients and the Emergency departments and 18 in the clinical wards. In 2024, 102 positive cultures were taken in the Outpatient and the Emergency departments and 21 in the clinical wards. There were patients from whom only one set of blood cultures was obtained, making the interpretation of the results challenging. Demographic data regarding the positive blood cultures between men and women for each year and their origin were recorded to detect possible differences in distribution between the two years of the present study. Analysis of the data for the years 2023 and 2024 revealed a stable distribution of gender and origin within the samples, with no statistically significant differences between the two years. Despite small fluctuations, the percentage of resistant *E. coli* strains did not show significant differences based on the origin (hospitalized or outpatient, as shown in Table 1.

### 3.1. Pathogen Distribution

For both years, Gram-positive cocci were more common than Gram-negative rods, with a total of 99 and 84 isolates, respectively. Of the Gram-positive cocci, 15 (15.15%) were *S. aureus*, 72 (72.72%) were coagulase-negative *Staphylococci*, and 12 (12.12%) were *Streptococci.* One strain of *S. aureus* was methicillin-resistant (MRSA). A strain of *Listeria monocytogenes* was also isolated. *Pediococcus pentosaceus* was isolated from a male patient in the Emergency Department from two different sets of blood cultures with the same biochemical reactions and antibiotic susceptibility patterns, and was considered a true pathogen.

Regarding *Enterococci*, two strains of *Enterococcus faecium* were isolated (one vancomycin-resistant, VRE) and six strains of *Enterococcus faecalis*. One strain each of *Streptococcus gallolyticus* and *Streptococcus pneumoniae* were also isolated. Of the Gram-negative rods, 65 (78.3%) were Enterobacterales. The frequency of isolation was, in descending order, *Escherichia coli*, *Klebsiella pneumoniae*, *Proteus mirabilis*, *Morganella morganii, Klebsiella aerogenes, Serratia species, Klebsiella oxytoca*, *Citrobacter freundii*, *Enterobacter cloacae,* and *Pantoea species*, as shown in Table 2.

The remainder comprised eight strains of *Pseudomonas aeruginosa*, four strains of *Acinetobacter baumannii* (one pandrug-resistant), three strains of *Stenotrophomonas maltophilia*, one strain of *Sphingomonas paucimobilis*, and one strain of *Campylobacter jejuni*. The isolated fungi comprised one strain of *Candida parapsilosis*, two sets from the same patient with *Candida glabrata*, and three sets of the same patient with *Candida tropicalis*.

Eight blood cultures were positive for two different microorganisms: a male patient in the Emergency Department with both *Enterococcus faecium* and *Klebsiella pneumoniae*, a female patient in the Internal Medicine ward with *Citrobacter koseri* and *Proteus mirabilis*, a male patient in the Emergency Department with *Candida tropicalis* and *Enterococcus faecalis* (the same patient had two more samples positive with *Candida tropicalis*), a male patient in the Emergency Department with *Staphylococcus epidermidis* and *Klebsiella pneumoniae*, a female patient in the Surgery Department with *Pseudomonas aeruginosa* and *Stenotrophomonas maltophilia*, a female patient in the Emergency Department with *Escherichia coli* and *Klebsiella pneumoniae*, a male outpatient with *Pseudomonas aeruginosa* and *Staphylococcus epidermidis* (the same patient had two more samples positive with *P. aeruginosa*), and a female patient in the Emergency Department with *Staphylococcus warneri* and *Staphylococcus haemolyticus*. Of the cases of coagulase-negative *Staphylococci*, only three were considered to be true bacteremia: a female patient in the Internal Medicine ward with one blood culture derived from central catheter and one from peripheral vein, both positive with the same strain to both biochemical reactions, and antibiotic susceptibility patterns of *S. epidermidis*; a female patient in the Emergency Department with two blood cultures collected from different peripheral veins, both positive for the same phenotypic strain of *S. epidermidis*; and a female outpatient with the same phenotypic strain, that is, with the same biochemical reactions and antibiotic susceptibility patterns of *Staphylococcus hominis*.

### 3.2. Antimicrobial Resistance

Regarding *Enterococci*, one strain was vancomycin-resistant (VRE). Of the total of 72 isolated strains of coagulase-negative *Staphylococci*, 43 (59.7%) were cefoxitin-resistant. Regarding *E. coli* strains, of the 38 isolated strains, 15 (39.5%) were resistant to ciprofloxacin. The distribution of *E. coli* strains resistant to ciprofloxacin was as follows: eleven from the Emergency Department and four from the Internal Medicine ward. Two of these strains were also extended-spectrum beta-lactamase-producing (ESBL+). Both of these strains originated from patients in the Emergency Department. All *E. coli* strains were tested for ESBL production. The resistance of *E. coli* strains to ciprofloxacin identified originating from both hospitalized patients and in the community revealed a non-statistically significant distribution (*p*-value 0.3666). A *p*-value less than 0.05 was considered significant. The group size for the inpatients is small, and this is a limitation of our study.

The pandrug-resistant strain of *Acinetobacter baumannii* originated from the Orthopedics Department and was a nosocomial strain. The patient was isolated, and all precautionary measures were applied. The *A. baumannii* strain was further tested for all relevant antibiotics by E-test to confirm pandrug resistance.

Pathogen distribution per ward and resistance rates, and pathogen, number of isolates, ward/origin, and key resistance markers are shown in Table 3 and Table 4, respectively.

### 3.3. True Bacteremia vs. Contamination Cases

Our data show quite a high number of blood cultures positive for coagulase-negative *Staphylococci*. It appears that the contamination rate in our hospital was 5.3%. Of the isolated CoNS, 41% were isolated from one bottle. The contaminants derived in descending order from the Hemodialysis Unit, Emergency Department, Internal Medicine ward, Surgery, and Orthopedics Department are shown in Table 3. Data regarding time-to-positivity (TTP) were not available. Data not provided to the laboratory staff included whether the Dialysis Unit drew cultures from peripheral veins, tunneled intravenous catheters, arteriovenous fistulas/grafts, or the hemodialysis circuit (the tubing connected to the catheter hub). The positivity rate for the Hemodialysis Unit was 32.4%; 64.3% of the isolated microorganisms were CoNS.

## 4. Discussion

Blood culture remains the gold standard for identifying the culprit bacteria or fungi isolated from bloodstream infections, which are a major cause of death both in the community and in hospitalized patients worldwide [1]. The isolation of the microorganism on agar plates is also important for antimicrobial susceptibility testing [1]. In this study, both an automated MIC measurement test and E-test were used to confirm resistance.

Sepsis not only impacts the likelihood of the best patient outcome, but also places demands on healthcare resources. According to the NHS, 40% of patient admissions are caused by bacterial infections, which are responsible for up to 66% of deaths [4]. Blood cultures are key to the early diagnosis and treatment of bacterial infections and sepsis, leading to improved antimicrobial stewardship [4]. It is critical to ensure that blood cultures are appropriately administered [1]; usually, they are performed when bacterial infection is suspected in patients who present with fever, elevated polymorphonuclear blood cell count and inflammation indices, and signs and symptoms of septic shock. These criteria also expand to incorporate immuno-compromised or elderly patients [1]. However, if the need for blood cultures is overestimated, this can lead to unnecessary burdens on laboratory staff and increasing costs, in addition to increasing the percentage of positive blood cultures up to 13%; 20–56% of these, according to published data, are due to contaminants. In our study, the percentage of positive blood cultures was 14.3%, generating immense workload and increasing cost on laboratory staff. All patients included in this study were febrile, but there was insufficient data regarding the signs and symptoms of septic shock in some cases.

The first step to accurate diagnosis is the elimination of pre-analytical mistakes. The risk of contamination is low when antisepsis procedures are implemented appropriately [5]; many methods of skin preparation prior to blood culture sample collection are referred to in the literature, with the common denominator being a dedicated phlebotomy team [6]. In healthcare settings where a dedicated phlebotomy team is not available, strict measures and staff education are necessary to improve blood sample collection. In recent studies, the alcoholic antiseptic chlorhexidine was found to be more effective than the non-alcoholic povidone–iodine (PVI) in blood sample collection [7,8]. The antiseptic agent should be applied to blood culture bottles prior to inoculation for optimal results, based on studies that have shown significantly less contamination [9,10]. The antiseptic used on a bottle varies according to the setting; some use alcohol-based antiseptics, whereas others use iodine solutions. If an iodine solution is used, it should be left to dry and then wiped off with alcohol [11]. In light of our findings, a standardized blood culture collection kit with chlorhexidine and isopropyl alcohol wipes, coupled with mandatory training for all clinical staff, was recommended to reduce the contamination rate.

In blood sampling, peripheral vein puncture is the optimal sampling site in comparison to drawing from available catheter lines to minimize the risk of contamination. If this is not possible, then discarding the first few milliliters (mL) of blood is recommended [5,8,12,13]. When a blood culture is derived from a previously installed vascular catheter, it is difficult to distinguish true bacteremia from contamination or colonization, if positive. In the literature, 15–25% of short-term central venous catheters were found to be colonized with coagulase-negative Staphylococci, the most frequent microorganisms [14,15]. Therefore, blood should only be drawn from central catheters if central-line-associated bloodstream infections are suspected. In these cases, if a blood culture originating from the central line catheter tests positive for the same phenotypic microorganism at least two hours prior to the peripheral venipuncture, the central line is most probably the source of bacteremia [16]. In our study, one female patient in the Internal Medicine ward, with one blood culture from the central line catheter and one from the peripheral vein, both positive for the same phenotypic strain of *S. epidermidis*, was considered to have true bacteremia.

Early clinical studies, including empirical data, concluded that a minimum of 20–30 mL of blood per set is required for optimal results [17,18,19]. These data are still valid, as bacterial or fungal density in the blood is low and therefore a positive culture requires the optimal volume of blood to be collected [20,21]. However, collecting more blood than set out in the manufacturer’s instructions leads to false positive results [22,23]. Each blood culture set includes a pair of bottles, one aerobic and one anaerobic, of which the aerobic bottle should be filled first. At least two sets are required [24]. Blood samples should be placed in the culture incubator immediately after sample collection; delays interfere with microbial growth and viability, and the temperature of the vial is affected by that of the environment. Therefore, good practice dictates that the minimum time from sample collection to incubation should not exceed four hours [4]. Bacteria will grow in the blood culture bottle when the environmental temperature is high and pre-incubation exceeds four hours. Under these conditions, the bacteria may already be in the stationary phase when the bottle is loaded into the incubator. Incubator algorithms cannot detect bacterial growth under these conditions, meaning that the samples may be falsely characterized as negative [25]. For pediatric patients, the optimal blood volume depends on age and weight, with 1–1.5 mL recommended for children weighing <11 kg and 7.5 mL for children weighing 11–17 kg [26]. Clear communication and collaboration among clinicians, sample transport providers, and laboratory staff is the only way to eliminate mistakes at this stage. All medical and nursing staff were invited to attend courses on the correct practice and handling of blood cultures. Additionally, detailed written instructions for sample collection were given to all departments and clinics.

Differentiation between contamination and true bacteremia should be improved using criteria such as the number of positive sets with the same phenotypic microorganism, the number of positive bottles in the same set, and isolation of the same microorganism from other biological fluids or sites of infection [1,27]. It is essential to eliminate contaminants of the blood culture pathway and optimize results by improving pre-analytical procedures [4]. The acceptable rates for blood culture contamination are up to 3% [2]. In our study, of the isolated CoNS, 41% were isolated from one bottle, strong evidence in favor of contamination. The contamination rate was 5.3%, higher than the 3% described in the literature.

At least two sets of blood cultures should be obtained from adult patients with no severe anemia to better estimate true infection. Non-fastidious bacteria isolated after more than five days of incubation may be contaminants [28,29,30,31,32,33]. Where obtaining two culture sets may be difficult due to significant blood loss, it is essential to consider the time to positivity. A previous study outlined that if the blood culture tests positive after less than 15 h of incubation, it is probably a true infection [34]. Time to positivity data were not available in our study.

The literature shows contradictory data regarding samples containing coagulase-negative *Staphylococci*, even when symptoms and signs of infection are present [35,36,37] and should be interpreted with caution in patients with prosthetic devices and central venous catheters [10,27,31,35,38]. In our study, three patients were cases of true bacteremia due to CoNS. The difficulty of distinguishing true infection from contamination when strains of coagulase-negative *Staphylococci* are isolated can lead to the improper use of vancomycin, unnecessary burden on laboratory staff, and increasing costs [39,40,41].

Bottles should be incubated for a maximum of five to seven days, even for fastidious bacteria, strict anaerobes, fungi, and *Brucella* [24,42]. Certain microorganisms, when isolated, should be considered indicative of true bacteremia, for example, *Staphylococcus aureus, Streptococcus pneumoniae*, Enterobacterales, *Pseudomonas aeruginosa*, and *Candida albicans* [35]. On the other hand, coagulase-negative *Staphylococci*, *Corynebacterium* species, *Bacillus* species other than *Bacillus anthracis, Cutibacterium acnes* (*Propionibacterium acnes*), and *Micrococcus* species are often considered contaminants, but should be interpreted as such with caution [35,36]. Coagulase-negative *Staphylococcus* strains represent most of all contaminants [9,35,38,41,43,44]. These data described in the literature are in accordance with our findings. The rates of true bacteremia due to coagulase-negative *Staphylococci* vary, but are reported to range from 10 to 25% [41]. For all other microorganisms isolated from blood cultures, merely relying on genus and species is not sufficient to reach a conclusion on the presence of true bacteremia [35].

The presence of more than one microorganism is often considered a sign of contamination, but when isolated from blood culture, this can denote true bacteremia and should be interpreted regarding the patient’s history and the clinical findings [45]. In some cases, two different microorganisms were isolated from the same patient in our study. In these cases, it is difficult to distinguish true infection from contamination, and therefore more than one set of blood cultures should be obtained.

The over-consumption of antimicrobials in Greece creates the appropriate high-selection pressure conditions for the emergence of resistant isolates [46]. Published data from Greece for the years 2010–2013 demonstrate the production of extended-spectrum β-lactamases (ESBLs) in 27.1% of Gram-negative isolates [47]. In recent studies, *E. coli* isolates have demonstrated ciprofloxacin resistance rates of up to 20–40% [48,49,50]. In blood-isolated strains containing extended-spectrum beta-lactamase-producing *Escherichia coli* and *Klebsiella pneumoniae*, the rate increased to 60% [51]. In our study, 39.5% of the *Escherichia coli* strains isolated were phenotypically resistant to ciprofloxacin. Moreover, 2 out of 38 *Escherichia coli* strains (5.2%) were ESBL-producing.

In our study, Gram-positive cocci were more common than Gram-negative rods, with a total of 99 and 83 isolates, respectively. This finding is not in accordance with former studies conducted in Greece that reveal Gram-negative microorganisms as the predominant pathogens [47]. Our finding that Gram-positive cocci were more common than Gram-negative rods is due to the high CoNS contamination rate and inclusion of hemodialysis outpatients in our study. The positivity rate for the Hemodialysis Unit was 32.4%; 64.3% of the isolated microorganisms were CoNS.

Our data suggests that *E. coli* remains the most common isolated microorganism among Gram-negative rods with increasing quinolone resistance. This finding is in accordance with similar studies from Greece [47]. The resistance of *E. coli* strains to ciprofloxacin originating from both hospitalized patients and in the community revealed a non-statistically significant distribution.

The best blood culture collection practices in a hospital without a dedicated phlebotomy team need educational courses and compliance monitoring. In this direction, prevention of contaminants should be pursued by the microbiology laboratory, infection control, nursing, information technology specialists, and administrative leaders [52].

## 5. Conclusions

The contamination rate in our study was quite high at 5.3%. As true bacteremia is still difficult to distinguish from contamination, in healthcare settings with no dedicated phlebotomy team, educational courses on the best practices for blood culture sampling and compliance monitoring are crucial. *E. coli* is the predominant pathogen among Enterobacterales. The resistance of *E. coli* strains to ciprofloxacin is an emerging threat, with the strains identified originating from both hospitalized patients and in the community with a non-statistically significant distribution.

## Figures and Tables

**Table 1:** Comparisons of gender and origin per year of patients with positive blood cultures in outpatients vs. hospitalized patients.

	*p*-Value	Result
Gender per year (Χ^2^)	0.9931	No statistically significant difference
Origin per year (Χ^2^)	0.0799	No statistically significant difference

Two-sided comparisons with a *p*-value less than 0.05 were considered significant.

**Table 2:** Pathogen distribution per sample and ward.

Pathogen	Hemodialysis Unit	Emergency Department	Internal Medicine Ward	Orthopedics Department	Surgery Department	Total
Gram-positive cocci						
*S. aureus*	7	4	3	1		15
CoNS	36	24	10	1	1	72
*E. faecalis*	3	2	1			6
*E. faecium*		2				2
*S. pneumoniae*		1				1
*Pediococcus pentosaceus*		2				2
*Streptococcus gallolyticus*		1				1
*Listeria*			1			1
*Campylobacter jejuni*		1				1
*Enterobacterales*						
*E. coli*	1	29	8			38
*K. pneumoniae*	1	6	4			11
*P. mirabilis*		4	2			6
*Morganella morganii*		2				2
*K. aerogenes*	1	1				2
*K. oxytoca*		1				1
*Citrobacter freundii*			1			1
*Enterobacter cloacae*		1				1
*Serratia* sp.			1	1		2
*Pantoea* spp.	1					1
*A. baumannii*		1	1	1	1	4
*Sphingomonas paucimobilis*	1					1
*Stenotrophomonas maltophilia*	2				1	3
*P. aeruginosa*	3	3	1		1	8
*Candida parapsilosis*			1			1
*Candida glabrata*		2				2
*Candida tropicalis*		3				3

**Table 3:** Pathogen distribution per ward and resistance rates.

Ward	*E. coli* Total Number of Samples (Resistant to Ciprofloxacin Samples)	*E. coli* ESBL Producers (Number of Samples)	CoNS: Total Number of Samples (Resistant to Cefoxitin Samples)	*S. aureus* MRSA Number of Samples	*E. faecium* VRE Number of Samples	*A. baumannii* Pandrug-Resistant Number of Samples	Total Blood Culture Samples Performed
Hemodialysis Unit	1 (not Ciprofloxacin-resistant)		36 (22 Cefoxitin-resistant)				111
Emergency Department	29 (11 Ciprofloxacin-resistant)	2	24 (14 Cefoxitin-resistant)		1		698
Internal Medicine Ward	8 (4 Ciprofloxacin-resistant)		10 (5 Cefoxitin-resistant)				401
Orthopedics Department			1 (1 Cefoxitin-resistant)	1		1	18
Surgery Department			1 (1 Cefoxitin-resistant)				52
Cardiology Department							10

**Table 4:** Pathogen, number of isolates, ward/origin, and key resistance markers.

Pathogen	Number of Isolates	Ward/Origin	Key Resistance Markers
*E. coli*	38	Hemodialysis Unit, Emergency Department, Internal Medicine Ward	Ciprofloxacin-resistant 39.5%, ESBL producers 5.2%
*S. aureus*	15	Hemodialysis Unit, Emergency Department, Internal Medicine Ward, Orthopedics Department	MRSA 6.67%
*E. faecium*	2	Emergency Department	VRE 50%
*A. baumannii*	4	Emergency Department, Internal Medicine Ward, Orthopedics Department, Surgery Department	Pandrug-resistant 25%

## Data Availability

The original contributions presented in this study are included in the article. Further inquiries can be directed to the corresponding author.
